# N-Acetylcysteine Supplementation Controls Total Antioxidant Capacity, Creatine Kinase, Lactate, and Tumor Necrotic Factor-Alpha against Oxidative Stress Induced by Graded Exercise in Sedentary Men

**DOI:** 10.1155/2011/329643

**Published:** 2011-08-23

**Authors:** Donrawee Leelarungrayub, Raphiphat Khansuwan, Prapas Pothongsunun, Jakkrit Klaphajone

**Affiliations:** ^1^Oxidative Stress, Nutrition and Exercise Biochemistry Laboratory, Department of Physical Therapy, Faculty of Associated Medical Sciences, Chiang Mai University, Chiang Mai 50200, Thailand; ^2^Sport Sciences, Graduate School, Chiang Mai University, Chiang Mai 50200, Thailand; ^3^Department of Rehabilitation Medicine, Faculty of Medicine, Chiang Mai University, Chiang Mai 50200, Thailand

## Abstract

Aim of this study was to evaluate the effects of short-term (7 days) N-acetylcysteine (NAC) at 1,200 mg daily supplementation on muscle fatigue, maximal oxygen uptake (VO_2max _), total antioxidant capacity (TAC), lactate, creatine kinase (CK), and tumor necrotic factor-alpha (TNF-**α**). Twenty-nine sedentary men (13 controls; 16 in the supplement group) from a randomized control were included. At before and after supplementation, fatigue index (FI) was evaluated in the quadriceps muscle, and performed a graded exercise treadmill test to induce oxidative stress, and as a measure of VO_2max _. Blood samples were taken before exercise and 20 minutes after it at before and after supplementation, to determine TAC, CK, lactate, and TNF-**α** levels. Results showed that FI and VO_2max _ increased significantly in the supplement group. After exercise decreased the levels of TAC and increased lactate, CK, and TNF-**α** of both groups at before supplementation. After supplementation, lactate, CK, and TNF-**α** levels significantly increased and TAC decreased after exercise in the control group. Whereas the TAC and lactate levels did not change significantly, but CK and TNF-**α** increased significantly in the supplement group. Therefore, this results showed that NAC improved the muscle fatigue, VO_2max _, maintained TAC, controlled lactate production, but had no influence on CK and TNF-**α**.

## 1. Introduction

Exercise is promoted for improving general health and preventing many diseases such as cardiovascular disease, diabetes mellitus, and cancer [1]. However, several studies have shown that short heavy exercise may induce adverse effects associated with oxidative stress in untrained humans through various pathways such as electron leakage within mitochondria, auto-oxidation of the catecholamine, NADPH activity, or an ischemic-reperfusion [2]. Oxidative stress induces oxidation processes in proteins, lipids, or DNA. Oxidation of all biological molecules is a result of organ dysfunction [3]. During muscle contraction with a short heavy load, numerous free radicals are produced that induce microinjury or an inflammation process and tumor necrotic factor-alpha (TNF-*α*), interleukin-6 (IL-6) [4], lactate dehydrogenase (LDH), and creatine phosphokinase (CPK) [5] are released. Thus, over-oxidative stress directly affects physical performance through the ability of muscles to contract to whole-body aerobic capacity [6]. 

Considerable interest has been shown in finding ways to prevent heavy exercise-induced free-radical production and muscle injury. Nutritional supplements, such as vitamin C (ascorbic acid) or vitamin E (alpha-tocopherol), have been very popular among athletes and individuals [7]. Pure glutathione (GSH), composed of glutamic acid, cysteine, and glycine, has been proposed to prevent oxidative stress, but its absorption and effectiveness in blood is low [8]. NAC is used clinically for patients with chronic obstructive pulmonary disease (COPD) or acute respiratory distress syndrome (ARDS). NAC supplementation over a short period (5 days) significantly decreased phagocytosis and improved the level of GSH in the blood of COPD patients [8, 9]. Furthermore, supplementation of NAC at low concentrations (600 mg) per day for 14 weeks reduced superoxide radicals and improved glutathione peroxidase in healthy volunteers, while a high dose at 1,200 mg per day significantly reduced hydrogen peroxide (H_2_O_2_) in COPD patients [9]. Interesting evidence showed that NAC inhibited muscle fatigue [10]. A previous study found that NAC delayed fatigue from repeated isometric handgrip maneuvers and inhibited glutathione oxidation in sedentary men after 150 mg/Kg of NAC had been ingested [11]. However, no studies had looked at the effect of NAC on muscle injury via CK and TNF-*α* using a graded exercise test. Thus, the aim of this study was to investigate the short-term effects of NAC supplementation on muscle fatigue and VO_2max _ for seven days at 1,200 mg per day and examine its ability to control CK, TNF-*α*, and lactate in sedentary men.

## 2. Materials and Methods

All programs were approved by the Human Ethics Committee (Declaration of Helsinki) at the Faculty of Associated Medical Sciences, Chiang Mai University, Thailand. All 36 volunteers were 20–24-year-old, nonsmoking, nonathletic, healthy males. Their body mass index (BMI) was all within normal range (18.5–24.9 kg/m^2^) according to the WHO and International Obesity Task Force. 

All volunteers were randomly allocated to control (*n*  =  18) or NAC supplement (*n*  =  18) groups. The supplement group took 600 mg of oral NAC (FLUIMUCIL A 600, ZAMBON Switzerland, Ltd.) twice per day after meals for 7 days, a total daily dose of 1,200 mg. Over the 7 days, the researchers telephoned the volunteers daily to ensure that they kept to regular diets and check if they had experienced side effects from the NAC supplementation such as headaches or abdominal pain. All experimental values for both groups were tested at two times: day 0 before supplementation began and day 8 after completing the 7-day course of supplementation.

All volunteers in both the control and NAC supplement groups participated in graded exercise on a mechanical treadmill on day 0 and day 8. The target heart rate (THR) was 85% of the maximal heart rate (MHR) (220-age (yr)) per individual (American College of Sport Medicine, ACSM; 2004) [12] and tested by following a graded exercise program. Blood samples were taken from all volunteers before exercise and after exercise following 20 minutes of quiet rest. Each 10 mL blood sample was taken from the anterior cubital vein of each volunteer and kept in an EDTA tube. Whole blood was separated to plasma for evaluation of CBC, TAC, lactate, and TNF-*α*. Serum CK was analyzed at the Central Laboratory at the Faculty of Medicine, Chiang Mai University, Thailand.

Isometric knee-extensor of quadriceps muscle was evaluated using a handheld dynamometer (Chatillon DMG-250, USA). Volunteers were positioned in a test chair, with a hip angle of 90 degrees, and the knee angle was fixed at 30 degrees vertically. The trunk, hip, and thigh were strapped down to avoid involuntary movements. The individual landmark for placing a hand-held dynamometer at the antero-inferior region of the leg was calculated from the distance between the tibial tuberosity and the superior aspect of the lateral malleolus, multiplied by 0.6 [13]. Sixty repetitive static knee extensions were performed, while maintaining the maximal force for three seconds each. The quadriceps fatigue index was calculated from the final 10 repetitions, divided by the initial 10, and multiplied by 100 [14]. 

All volunteers warmed up by stretching large muscle groups of the upper and lower extremities for three minutes. Heart rate was recorded using a Polar heart rate monitor (F11, USA). A graded exercise test was performed on a mechanical treadmill, with the eight steps of a modified Bruce protocol (three minutes for each step as follows: step I: speed 1.7 mph with 0% slope, step II: speed 1.7 mph with 5% slope, step III: speed 1.7 mph with 10% slope, step IV: speed 2.5 mph with 12% slope, step V: speed 3.4 mph with 14% slope, step VI: speed 4.2 mph with 16% slope, step VII: speed 5.0 mph with 18% slope, and step VIII: speed 5.5 mph with 20% slope) [15]. A rate of perceived exertion (RPE) of 15 (hard) on a 20-point scale was the limit set for stopping exercise as well as showing physical signs such as muscle pain, cramp, headaches, heart palpitations, heartburn, blurred vision, or severe dyspnea (American College of Sport Medicine's Guidline, 2004) [12]. Predicted VO_2max _ was calculated for each volunteer, using the final speed and grade of the treadmill [16].

Total antioxidant capacity (TAC) of fresh plasma was assayed with ABTs cation radical decolorization [17]. Stock ABTs cation radical was produced by mixing ABTs (14 mmoL/L) and potassium persulfate (14 mmoL/L) together, and leaving in the dark overnight. Working ABTs cation radical was diluted in deionized water until absorbance was shown between 0.68 and 0.74 at 734 nm, before adding plasma. 10 *μ*L of plasma were added to 990 *μ*L of working ABTs cation radical and gently alternated inversely by shaking nine times before adding in the spectrophotometer. Decreased absorbance was recorded continuously every minute for three minutes and finally calculated to ΔA/min. The TAC of the plasma was calculated by comparing with the ΔA/min of standard *N*-acetylcysteine (Sigma) (0–10 mmoL/L).

Lactate was detected following the original protocol of Khantipongse [18] with a high-performance liquid chromatography (HPLC) system. The lactate was detected by UV wavelengths at 240 nm and separated in a C-18 HPLC column (Phenomenex, USA) (250  ×  4 mm, 5 *μ*m), with a mobile phase comprising a mixture of 0.05 mol/L NaH_2_PO_4_ and acetonitrite 1% (99 : 1%, v/v) (pH 2.4) at a 1 mL/min flow rate. The lactate concentration was compared with standard lactate (Sigma) at 0–20 mmoL/L.

The concentration of TNF-*α* in plasma was determined by following the guideline manual (Quantikine, R&D Systems, Inc, Minneapolis, MN, USA). Two hundred microliters of plasma and 50 mL of external standard TNF-*α* (50 pg/mL) were loaded in the anti-TNF-*α* polyclonal antibody-immobilized solid phase and incubated for two hours at room temperature. After being washed three times with wash buffer at 400 *μ*L, 200 *μ*L of HRP-conjugated TNF-*α* was added in each well, then, incubated for two hours at room temperature. All solutions in each well were aspirated and washed with wash buffer three times before adding 400 *μ*L of substrate TMB and incubating for 20 minutes at room temperature. Finally, 50 *μ*L of stop solution (2N sulfuric acid) was added, the blue color changed to yellow, and the absorbance was detected at 450 nm within 30 minutes by a microplate reader. Concentration of plasma TNF-*α* was calculated by comparing the plasma absorbance to the standard IL-2's absorbances (15.6–1,000 pg/mL). 

All parameters (before and after exercise, at day 0 and day 8, resp.) were analyzed statistically with repeated measurements in the General Linear Model (2  ×  2 factors) using a Least Significant Difference (LSD) test. Values were mean ±SE. Significance was set at *P* < .05.

## 3. Results

There was no difference in BMI or screening CBC characteristics of all volunteers at day 0 in the control and NAC supplement groups (Table 1). Results from the CBC, including WBC, RBC, Hb, Hct, and Plt in both groups, showed a healthy, normal status. Of the original 36 volunteers, 29 completed the study (13 controls and 16 in the NAC supplement group). Predicted maximal heart rate (PreMHR) was not different between the control (199.38 ± 1.78 bpm) and supplement (199.31 ± 0.19 bpm) groups and calculated target heart rate (THR) in the two groups was nearly identical (169.47 ± 1.52 and 169.40 ± 0.16 bpm). At day 0 and day 8, resting HR in the control group (86.85 ± 3.11 and 94.00 ± 2.74 bpm, *P* = .017) was significantly different, but not in the supplement group (85.68 ± 2.80 via 88.31 ± 2.47 bpm, *P* = .311). With exercise, the THR in the control group at day 0 was 169.54 ± 2.36 bpm, and 172.31 ± 1.21 bpm at day 8, whereas that in the supplement group at day 0 was 169.12 ± 2.13 bpm, and 172.94 ± 1.09 bpm at day 8, which showed no significant difference between or within groups. However, the RPE in the supplement group was significantly lower (14.37 ± 0.31 at day 0 and 13.06 ± 0.34 at day 8) when compared to the control group (13.46 ± 0.34 at day 0 and 13.77 ± 0.37 at day 8). Nevertheless, comparison of RPE between both groups at day 8 showed no significant difference. 

The percentage fatigue index (% FI) (Table 2) of the dominant quadriceps muscle in the control and supplement groups showed no significant difference (80.50 ± 3.11 and 81.42 ± 2.90%, *P* = .834) at day 0 (note that the higher the % FI, the less the fatigue). After supplementation for 7 days, the % FI for the supplement group increased more than (from 81.42 ± 2.99 to 90.67 ± 2.07 %, *P = .*009) that in the controls (80.51 ± 3.11 to 86.07 ± 2.25%, *P = .*140). There was no significant difference in VO_2max _ between the two groups at day 0 (36.03 ± 1.75 and 38.32 ± 1.58 mL/kg/min in the control and supplement group, resp.). Nonetheless, after supplementation for 7 days, the supplement groups showed a significant increase in VO_2max _ (38.32 ± 1.58 to 41.14 ± 1.35 mL/kg/min, *P = .*009) when compared to the control group (36.03 ± 1.75 to 34.58 ± 1.49 mL/kg/min, *P = .*201).

For the blood markers (Table 3), the baseline TAC at day 0 was 1.69 ± 0.07 mmoL NAC/L and 1.58 ± 0.06 mmoL NAC/L (*P = .*269) in the control and supplement groups, respectively. At day 8, prior to exercise, the baseline TAC was 1.73 ± 0.02 mmoL NAC/L in the control group (with no significant difference from day 0 (*P = .*584)) and 1.71 ± 0.02 mmoL NAC/L in the supplement group (*P = .*296), which was not different from the control group levels. However, results of TAC decreased significantly after the exercise test in the control group at day 0 (1.69 ± 0.07 to 1.49 ± 0.06 mmoL NAC/L, *P = .*000) and day 8 (1.73 ± 0.02 to 1.63 ± 0.03 mmoL NAC/L, *P = .*006), and in the supplement group at day 0 only (1.58 ± 0.06 to 1.38 ± 0.05 mmoL NAC/L, *P = .*005). Conversely, after NAC supplementation for 7 days, the TAC level did not reduce after exercise (1.71 ± 0.02 to 1.72 ± 0.03 mmoL NAC /L, *P = .*655), despite there is no difference in value at baseline compared to the control. 

Levels of CK increased after exercise in the control group at day 0 (148.8 ± 14.0 to 164.7 ± 16.1 U/L, *P = .*032) and day 8 (147.7 ± 17.8 to 163.9 ±  20.1 U/L, *P = .*015), and in the supplement group at day 0 (176.4 ± 16.7 to 190.5 ± 14.3 U/L, *P = .*035) and day 8 (137.7 ± 16.0 to 155.4 ± 18.1 U/L, *P = .*003). When comparing between groups at day 0 (before, *P = .*157, after exercise, *P = .*244) or day 8 (before, *P = .*681, after exercise, *P = .*756), there were no significant differences. 

At baseline at day 0 and 8, the levels of lactate in the control group (1.96 ± 0.35 and 2.01 ± 0.47 mmoL/L) were no different from those in the supplement group (1.91 ± 0.32, *P = .*919 and 2.05 ± 0.56, *P* = 0.982). Exercise increased lactate at day 0 in the control group (1.96 ± 0.35 to 3.67 ± 0.73, *P = .*049) and the supplement group (1.91 ± 0.32 to 3.72 ± 0.56, *P = .*029), as well as at day 8 in the control group (2.01 ± 0.47 to 3.84 ± 0.61, *P = .*034). However, after 7 days of supplementation, the lactate level did not increase significantly (2.05 ± 0.56 to 2.75 ± 0.55, *P = .*353) in the supplement group when compared to the control group. 

TNF-*α* levels at baseline (day 0) in the control (1.76 ± 0.22 pg/mL) and supplement group (1.86 ± 0.19 pg/mL) (*P = .*157) were similar. In addition, no significant difference was noted between the control and supplement groups at baseline at day 8 (1.40 ± 0.15 pg/mL and 1.38 ± 0.13 pg/mL, *P = .*915). After exercise, TNF-*α* significantly increased in the control group (day 0: 1.76 ± 0.22 to 5.71 ± 0.56 pg/mL, *P = .*000; day 8: 1.40 ± 0.15 to 5.68 ± 0.52 pg/mL, *P = .*000) and supplement group (day 0; 1.86 ± 0.19 to 6.93 ± 0.51 pg/mL, *P = .*000 and day 8; 1.38 ± 0.13 to 5.06 ± 0.47 pg/mL, *P = .*000). No differences between groups were noted in this response (*P = .*385).

## 4. Discussion

This study used NAC to replace pure GSH because previous evidence showed that oral supplement of pure GSH at 3.0 mg had low absorption and no significance when maintained in plasma and compared to the control group [19]. Orally-administered NAC at 600 mg in humans resulted in a mean maximum plasma concentration of L-cysteine of 4.6 *μ*mol/L and a mean cysteine concentration from 10 *μ*mo/L to a maximum level of 18.6 *μ*mol/L after one hour [20]. In the pharmacokinetics of NAC after oral administration in a rat model, 77% was maintained in the body with 3% extracted in feces, and the major metabolites as cysteine and cystine in the liver and inorganic sulphate were urinary excretion products [21]. However, a previous study showed that a high concentration of NAC (1.8 g daily for 2 weeks) increased plasma cysteine but did not improve the plasma GSH significantly [22]. Therefore, it is possible that despite increases in cysteine, the antioxidant effects of GSH might not be realized. 

In a previous evaluation [23], high reliability of fatigue measurements could be calculated from 50 maximal concentric contractions performed on a dynamometer. However, the fatigue index in this study was modified by using 60 repetitive contractions, as all volunteers were healthy young men. The data showed that the resting percentages of fatigue index in the control group at day 0 and day 8 were not significantly different; however values for the supplement group improved from day 0 to day 8 (Table 2). 

Previous evidence has shown that lactate does not affect muscle fatigue, but inorganic phosphate from creatine phosphate was the main cause of it [24]. Furthermore, muscle fatigue may be related to increases in free radicals in skeletal muscle fibers. Early work has demonstrated that the free radicals scavenger, NAC, delays muscle fatigue [25]. In this study, a significant change of 81.42 ± 2.99% at day 0 to 90.67 ± 2.07% at day 8 was noted in the fatigue index within the supplement group at day 8 after 7 days of NAC supplementation. 

In addition to the fatigue index, estimated VO_2max _ increased with NAC treatment. At day 0, the VO_2max _ level of the supplement group showed no difference from that in the control group. After 7 days of supplementation, the VO_2max _ level increased (at day 8) in the supplement group, when compared to the control group. Taken collectively, it appears that NAC may provide an ergogenic benefit, but the results were mixed. 

This study did not evaluate the NAC in plasma, but a previous report showed that the level of cysteine in NAC increased after orally supplementing NAC at 400 mg per single dose to 4 mg/L in plasma [26]. The results showed that the TAC in the supplement group increased after supplementation at day 8, when compared to day 0, but with no significant change. The results showed the benefit of NAC in maintaining the TAC levels, which were found to be effective in protecting against oxidative stress after graded exercise, when compared with the control group. 

Exercise that induces systemic inflammation by releasing many cytokines such as interleukin-6 (IL-6), interleukin-1 (IL-1), interleukin-10 (IL-10), TNF-*α*, and CK is related to muscle damage [27, 28]. This study showed a significantly high concentration of CK after exercise in both groups at day 0 and day 8. TNF-*α* increased significantly in both groups at day 0 and day 8. No effect was observed with NAC supplementation, while in a previous study on NAC supplementation, a longer period of treatment (3 months) showed a significant decrease in TNF-*α* [11]. Lactate increases in an intensity-dependent manner with exercise, possibly from 1.5 mmoL/L at rest to 12.3 mmoL/L after running [29]. It also presents within blood 20 minutes after exercise and returns to resting level within two hours [30]. This study detected plasma lactate after 20 minutes exercise and results showed a significant increase in the control and supplement groups. After NAC supplementation for 7 days, the lactate response to exercise was less than that at day 0, suggesting the possible benefit of NAC supplementation.

Overall results indicated that NAC at 1,200 mg daily can reduce oxidative stress from short exercise. However, the contrast in application still needs more proof because low levels of free radicals that generate in mitochondria are very important for normal muscle force, and they increase more with stronger force [31]. Previous reports suggested that antioxidants such as vitamin C and vitamin E might inhibit the defense mechanism or adaptive response from exercise-induced free radicals [32]. In addition, supplementation of vitamin C for 8 weeks improved VO_2max _ in trained men, but there were adverse effects from expression reduction of key transcription factors such as Mn-SOD and PGx in a rat model [33]. Finally, much evidence has recommended that supplementation of antioxidant affected cellular adaptation by either downregulation or upregulation pathways. This study also needed more specific evidence of NAC showing the benefits or adverse effects between low- and high-dose supplementation in the future.

## 5. Conclusion

This study shows that supplementation of FLUIMUCIL, which contains mainly NAC, at 1,200 mg daily for 7 days, helps to protect from muscle fatigue and may maintain TAC following strenuous exercise. However, it does not influence CK and TNF-*α* release.

## Figures and Tables

**Table 1:** Characteristics and complete blood count data at day 0 for volunteers in both the control and supplement group.

	Control (*n* = 13)	NAC (*n* = 16)
Aged (yr)	19.49 ± 3.58	20.01 ± 0.79
	(18–22)	(18–23)
BMI (kg/m^2^)	21.14 ± 2.71	21.34 ± 3.14
	(17.5–28.4)	(18.3–30.5)
WBC (10^3^/*μ*L)	6.83 ± 1.19	6.04 ± 1.15
	(4.2–9.0)	(4.8–7.9)
RBC(10^3^/*μ*L)	5.68 ± 0.60	5.49 ± 0.39
	(4.9–6.6)	(4.97–6.19)
Hb(gm/dL)	14.65 ± 1.37	15.45 ± 0.7
	(11.7–16.8)	(14.1–16.4)
Hct (%)	45.31 ± 3.5	47.48 ± 2.13
	(37.1–50.9)	(43.7–50.9)
Plt (10^3^/*μ*L)	318.84 ± 64.7	246.5 ± 49.68
	(185–428)	(155–354)

Values are mean ± SD and (Min-Max). The values represent the range.

**Table 2:** Fatigue index (%) and VO_2 max_ (mL/kg/mL) at pre- and postexercise test between the control and supplement groups.

	Control (*n* = 13)		NAC (*n* = 16)	
	Pre-ex	Post-ex	Pre-ex	Post-ex
Fatigue index	80.50 ± 3.11	86.07 ± 2.19	81.42 ± 2.99	90.67 ± 2.07*
	(62.21–99.38)	(71.84–96.35)	(53.80–97.22)	(75.94–99.65)
VO_2max _	36.03 ± 1.75	34.58 ± 1.49	38.32 ± 1.58	41.14 ± 1.35*
	(24.14–52.01)	(24.13–42.22)	(33.21–52.01)	(33.02–52.01)

Values are mean ± SE (Min-Max). The values represent the range. *P < 0.05 compared to pre-ex period within the group.

**Table 3:** Total antioxidant capacity (TAC), lactate (mmoL/L), creatine kinase (U/L), and tumor necrotic factor-alpha (TNF-*α*) (pg/mL) between the control and supplement groups at day 0 and 8.

	Control (*n* = 13)				NAC (*n* = 16)			
	day 0		day 8		day 0		day 8	
	Pre-ex	Post-ex	Pre-ex	Post-ex	Pre-ex	Post-ex	Pre-ex	Post-ex
TAC	1.69 ± 0.07	1.49 ± 0.06^#^	1.73 ± 0.02	1.63 ± 0.03^#^	1.58 ± 0.06	1.38 ± 0.05^#^	1.71 ± 0.02	1.72 ± 0.03
	(1.26–1.89)	(1.10–1.41)	(1.65–1.80)	(1.51–1.77)	(0.83–1.88)	(0.74–1.81)	(1.66–1.86)	(1.53–1.94)
Lactate	1.96 ± 0.35	3.67 ± 0.73^#^	2.01 ± 0.47	3.84 ± 0.61^#^	1.91 ± 0.32	3.72 ± 0.56^#^	2.05 ± 0.56	2.75 ± 0.55
	(0.56–4.78)	(0.34–7.45)	(0.11–6.12)	(0.53–6.67)	(0.11–1.21)	(0.11–11.30)	(0.45–6.12)	(0.23–6.34)
CK	148.8 ± 14.0	164.7 ± 16.1^#^	147.7 ± 17.8	163.9 ± 20.1^#^	176.4 ± 12.7	190.5 ± 14.3^#^	137.7 ± 60.0	155.4 ± 18.1^#^
	(89–282)	(90–290)	(87–310)	(95–372)	(123–285)	(111–301)	(56–248)	(95–290)
TNF-*α*	1.76 ± 0.22	5.71 ± 0.56^#^	1.40 ± 0.15	5.68 ± 0.52^#^	1.86 ± 0.19	6.93 ± 0.51^#^	1.38 ± 0.13	5.06 ± 0.47^#^
	(0.78–3.12)	(4.81–8.45)^#^	(0.78–2.34)	(2.56–7.65)	(0.87–3.56)	(3.42–9.89)	(0.67–2.12)	(2.12–9.48)

Values are mean ± S.E (Min-Max). The values represent the range. ^#^ *P* < 0.05 compared to pre-exercise (pre-ex) period on the same day.
